# Supplementary material for: Validation of performance of Spanish version of PURE-4 questionnaire for early identification of psoriatic arthritis after 1 year of follow-up in patients with psoriasis
Source: PLoS One. 2026 Mar 2;21(3):e0342498. doi: 10.1371/journal.pone.0342498 (PMC12952629; doi:10.1371/journal.pone.0342498)
Supplement: S1 Table — FPFV, First Patient First Visit; LPLV, Last Patient Last Visit; SIV, Site Initiation Visit. (DOCX) [file pone.0342498.s001.docx]

**S1 Table.** Sites and study recruitment data

| **Site** | **SIV** | **FPFV** | **LPLV** |
| --- | --- | --- | --- |
| HUCA de Oviedo | February 22, 2021 | March 4, 2021 | July 22, 2022 |
| Hospital General de Alicante | March 8, 2021 | March 16, 2021 | May 31, 2022 |
| Hospital Moises Broggi | February 10, 2021 | February 12, 2021 | June 29, 2022 |
| Hospital Sant Pau | February 12, 2021 | March 12, 2021 | July 11, 2022 |
| Hospital del Mar | January 15, 2021 | January 28, 2021 | June 23, 2022 |
| Hospital 12 de Octubre | December 23, 2020 | January 21, 2021 | June 8, 2022 |
| Hospital Infanta Leonor | February 2, 2021 | March 1, 2021 | April 22, 2022 |
| Hospital Virgen del Rocio | March 24, 2021 | April 8, 2021 | June 17, 2022 |
| Hospital de Valme | May 31, 2021 | June 8, 2021 | September 13, 2022 |
| Hospital Vall Hebron | February 26, 2021 | March 26, 2021 | July 6, 2022 |
| Hospital La Fe | February 10, 2021 | February 17, 2021 | June 15, 2022 |
| Hospital de Meixoeiro | March 11, 2021 | March 18, 2021 | October 7, 2022 |
| Hospital de Santiago | December 21, 2020 | March 2, 2021 | August 2, 2022 |
| Hospital Ramón y Cajal | March 10, 2021 | June 23, 2021 | May 10, 2022 |
| Hospital de Basurto | April 8, 2021 | May 14, 2021 | July 12, 2022 |
| Hospital de La Paz | February 23, 2021 | March 17, 2021 | August 24, 2022 |
| Hospital Universitario de Tenerife | April 20, 2021 | June 23, 2021 | September 13, 2022 |
| Hospital de Manacor | December 18, 2020 | December 22, 2020 | May 3, 2022 |
| Hospital de Getafe | February 26, 2021 | March 1, 2021 | August 19, 2022 |

FPFV, First Patient First Visit; LPLV, Last Patient Last Visit; SIV, Site Initiation Visit.
