# Supplementary material for: Validation of performance of Spanish version of PURE-4 questionnaire for early identification of psoriatic arthritis after 1 year of follow-up in patients with psoriasis
Source: PLoS One. 2026 Mar 2;21(3):e0342498. doi: 10.1371/journal.pone.0342498 (PMC12952629; doi:10.1371/journal.pone.0342498)
Supplement: S2 Table — BSA, body surface area; DLQI, Dermatology Life Quality Index; n, number of patients (unless otherwise specified); PASI, Psoriasis Area and Severity Index; PGA, Physician Global Assessment; PsA, psoriatic arthritis; SD, standard deviation. (DOCX) [file pone.0342498.s002.docx]

**S2 Table.** Baseline clinical and sociodemographic characteristics of all evaluable psoriasis patients enrolled in Assessment I and Assessment II (variables collected in Assessment I)

| **Characteristics** | **With diagnosis of PsA (at the end of the study)**  **(n = 46)** | **Without diagnosis of PsA (at the end of the study)**  **(n = 205)** | **Non-evaluable (at the end of the study)**  **(n = 2)** | **Total**  **(n = 253)** | **P-value** |
| --- | --- | --- | --- | --- | --- |
| Age, years, mean (SD) | 48.9 (11.1) | 47.0 (12.5) | 39.0 (19.8) | 47.3 (12.3) | 0.3365 |
| Male, n (%) | 28 (60.9) | 115 (56.1) | 0 | 143 (56.5) | 0.5547 |
| Years from the diagnosis of psoriasis, mean (SD) | 20.4 (14.2) | 18.7 (12.8) | 13.1 (9.8) | 18.9 (13.0) | 0.5290 |
| Special sites of psoriasis, n (%) | 31 (67.4) | 95 (46.3) | 1 (50.0) | 127 (50.2) | 0.0099 |
| Patients on treatment for psoriasis, n (%) | 40 (87.0) | 182 (88.8) | 2 (100.0) | 224 (88.5) | 0.7265 |
| PASI, mean (SD) | 8.7 (5.6) | 6.8 (5.0) | 9.5 (3.5) | 7.2 (5.1) | 0.0263 |
| Mild (PASI <7), n (%) | 14 (30.4) | 93 (45.4) | 0 | 107 (42.3) | 0.0642 |
| Moderate/severe (PASI ≥7), n (%) | 32 (69.6) | 112 (54.6) | 2 (100.0) | 146 (57.7) |  |
| BSA, mean (SD) | 10.8 (8.3) | 8.2 (8.1) | 5.0 (-) | 8.7 (8.2) | 0.0624 |
| Valid n | 3 | 40 | 1 | 44 |  |
| PGA | | | | | |
| Valid n | 42 | 158 | 1 | 201 |  |
| 0. Clear: No signs of psoriasis (post-inflammatory hyperpigmentation may be present), n (%) | 3 (7.1) | 14 (8.9) | 0 | 17 (8.5) | 0.0611 |
| 1. Nearly clear, minimal: Minimal plaque elevation, scaling and/or erythema, n (%) | 4 (9.5) | 35 (22.2) | 0 | 39 (19.4) |  |
| 2. Mild: Mild plaque elevation, scaling and/or erythema, n (%) | 9 (21.4) | 46 (29.1) | 1 (100.0) | 56 (27.9) |  |
| 3. Moderate: Moderate plaque elevation, erythema and/or scaling, n (%) | 17 (40.5) | 49 (31.0) | 0 | 66 (32.8) |  |
| 4. Severe: Very marked plaque elevation, erythema and/or scaling, n (%) | 9 (21.4) | 14 (8.9) | 0 | 23 (11.4) |  |
| DLQI, mean (SD) | 9.9 (6.9) | 7.6 (6.7) | 20.0 (-) | 8.2 (6.9) | 0.0938 |
| Valid n | 16 | 87 | 1 | 104 |  |

BSA, body surface area; DLQI, Dermatology Life Quality Index; n, number of patients (unless otherwise specified); PASI, Psoriasis Area and Severity Index; PGA, Physician Global Assessment; PsA, psoriatic arthritis; SD, standard deviation.
