# Supplementary figures and images for: Validation of performance of Spanish version of PURE-4 questionnaire for early identification of psoriatic arthritis after 1 year of follow-up in patients with psoriasis
Source: PLoS One. 2026 Mar 2;21(3):e0342498. doi: 10.1371/journal.pone.0342498 (PMC12952629; doi:10.1371/journal.pone.0342498)

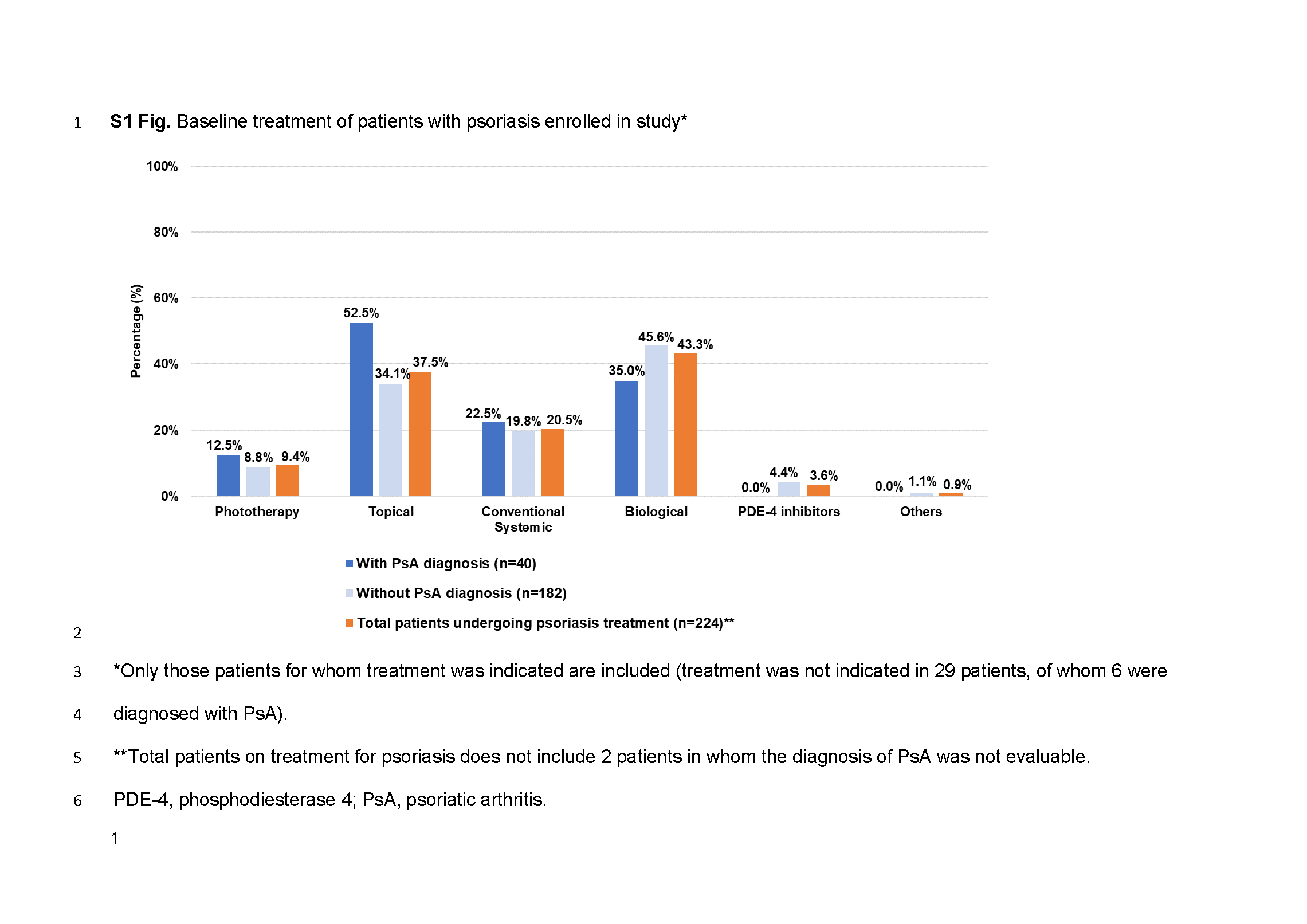

Supplement: S1 Fig — *Only those patients for whom treatment was indicated are included (treatment was not indicated in 29 patients, of whom 6 were diagnosed with PsA). **Total patients on treatment for psoriasis does not include 2 patients in whom the diagnosis of PsA was not evaluable. PDE-4, phosphodiesterase 4; PsA, psoriatic arthritis. (TIFF) [file pone.0342498.s003.tiff]

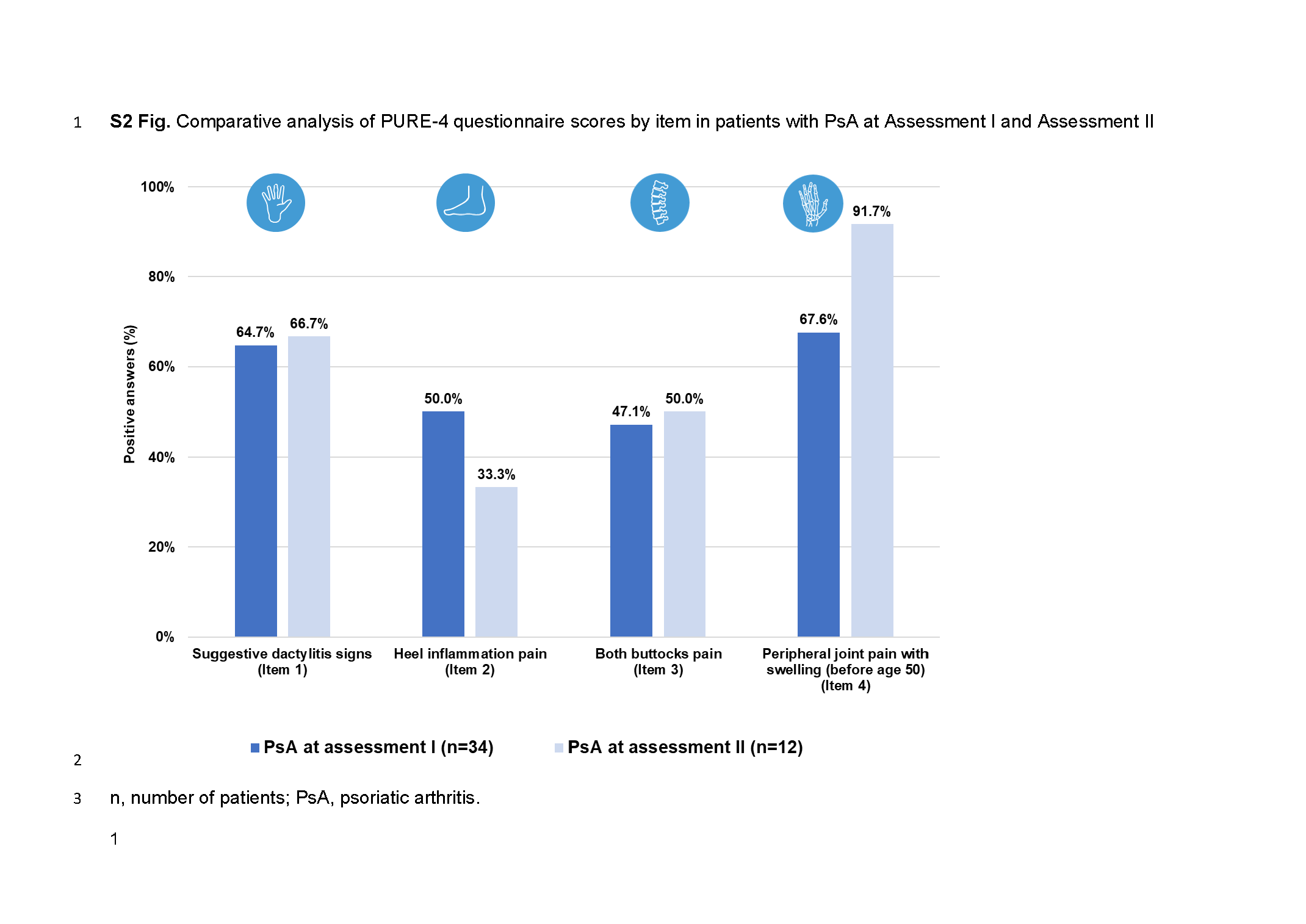

Supplement: S2 Fig — n, number of patients; PsA, psoriatic arthritis. (TIF) [file pone.0342498.s004.tif]
