# Supplementary material for: Validation of performance of Spanish version of PURE-4 questionnaire for early identification of psoriatic arthritis after 1 year of follow-up in patients with psoriasis
Source: PLoS One. 2026 Mar 2;21(3):e0342498. doi: 10.1371/journal.pone.0342498 (PMC12952629; doi:10.1371/journal.pone.0342498)
Supplement: Attachment — Submitted filename: Plos one.docx [file pone.0342498.s005.docx]

1. The background introduction of PURE-4 is too brief. Could you provide a more detailed introduction?
2. Could you list the detailed items of the PURE-4 questionnaire?
3. It is noted that 219 participants were included in the study, but the final ROC curve was plotted with n=217. Could you explain why two people were excluded?
4. Could the ROC curve be included in the manuscript?
5. The baseline data for psoriatic arthritis are too sparse. Could some interesting indicators be added to enrich the content?

6. The manuscript mainly describes the specificity and sensitivity of PURE-4 in predicting the development of psoriatic arthritis one year later. Compared with other predictive indicators, does it have an advantage?
