# Supplementary material for: Validation of performance of Spanish version of PURE-4 questionnaire for early identification of psoriatic arthritis after 1 year of follow-up in patients with psoriasis
Source: PLoS One. 2026 Mar 2;21(3):e0342498. doi: 10.1371/journal.pone.0342498 (PMC12952629; doi:10.1371/journal.pone.0342498)
Supplement: Attachment — Submitted filename: Answers to reviewers comments_PURE-4 questionnaire.docx [file pone.0342498.s009.docx]

Dear Academic Editor,

Thank you for your letter and constructive comments concerning our manuscript entitled ‘*Validation of performance of Spanish version of PURE-4 questionnaire for early identification of psoriatic arthritis after 1 year of follow-up in patients with psoriasis*'.

We have carefully reviewed your comments and have revised the manuscript accordingly. Our responses are given in blue in a point-by-point manner below. Changes to the documents are implemented in “track-changes” and we have submitted two revised versions, one with tracked-changes and one clean version without tracked-changes, for ease of your review.

We trust that the revised version is now suitable for publication and look forward to your feedback in due course.

Yours sincerely,

Rubén Queiro-Silva

# **Response to Academic Editor and Reviewers**

Thank you for considering our work and for the thorough revision. We have paid attention to all your comments and answered them to clarify all the points that were raised. We have also amended the manuscript aligned with your comments (if applicable). We hope that now this version of the manuscript will be considered for publication.

We greatly appreciate your time and consideration.

# **Response to Academic Editor**

***Title and Abstract***

- Briefly describe psoriatic arthritis as a disease.

We appreciate the Academic Editor's observation regarding the lack of information about the description of the disease. Considering it, we have included additional information about it in the first sentence of the Background and objective subsection of the Abstract.

Also, we have implemented the necessary changes to the abbreviations in the same subsection of the Abstract.

- Briefly give the key findings of your study. Include key numeric data (including confidence intervals or *p* values)

We have added some numerical information according to the Academic Editor’s comment. However, we think key findings and key numeric data are included in the current Abstract, so we propose maintaining it as it appears in the modified version.

***Introduction***

- Briefly describe psoriatic arthritis as a disease.

Again, we thank the Academic Editor and, according to his/her comment, we have included additional information about the description of the disease in the first sentence of the Introduction section of the manuscript.

Aligned with the introduction to this information, we have implemented the necessary changes to the abbreviations and references in the full text of the manuscript.

- The gaps in knowledge and rationale for the study need to be mentioned.

We express our gratitude to the Academic Editor for highlighting the importance of addressing the existing gaps and for bringing this issue to our attention. In response to his/her feedback, we have implemented minor but strategic changes to enhance clarity and comprehension in this section of the manuscript.

***Methods***

- Please outline this section following the STROBE guidelines.

We have meticulously reviewed both the Materials and methods section, as well as the entire manuscript, and can confirm that they adhere to the key elements of the STROBE guidelines, which are structured according to the STROBE guidelines for cross-sectional studies [^[1]^].

- You need to state in the Methods section that you have followed relevant EQUATOR guidelines. e.g., STROBE: “The reporting of this study conforms to STROBE guidelines”. (Insert new reference number, Note, you will probably need to renumber your references after this addition).

Thank you very much for bringing this to our attention. We have included the phrase "The reporting of this study conforms to STROBE guidelines" at the end of the Study Design subsection within the Materials and methods section. Additionally, we have referenced this phrase [1] and renumbered the necessary references, accordingly, as indicated by the Academic Editor.

- Need subheading for more clarity

We thank the reviewer for pointing it out. As indicated by the editor, we have included the subheading "Study assessment" to improve the clarity of the Materials and methods section.

- How were the patients selected (e.g., consecutively, randomly, or selectively)?

For Assessment I, patients were consecutively included based on clinical practice, as they met all the inclusion criteria and none of the exclusion criteria. The inclusion criteria were reported in a previously published article [^[2]^], which is referenced in the present manuscript in the Study Design subsection within the Materials and methods section. All patients who were not diagnosed with psoriatic arthritis at Assessment I were eligible for a second rheumatology visit one year later (±2 months) to confirm or rule out the presence of PsA one year after completing the PURE-4 questionnaire.

In order to clarify this point further, we have expanded the explanation whenever we have deemed it appropriate throughout the Materials and methods section.

- Describe how the study size was arrived at.

Thank you very much for highlighting the importance of the study size. The explanation regarding how the study size was determined is provided in the fourth paragraph of the Statistical Analysis subsection within the Materials and methods section. However, if you require further information or would like us to elaborate on any particular aspect, we are more than willing to do so.

***Discussion***

- In the grand scheme of things, please attempt to summarize key findings in relation to the study's objectives.

We are sincerely grateful for the suggestion. However, after carefully reviewing the Discussion section, we believe that, in its current form, it adequately summarizes the key findings of our study in relation to the stated objectives. Nonetheless, we remain open to making further adjustments should the journal require it or if specific aspects are identified that could be improved for greater clarity.

- Have you compared your results with relevant previous papers, and cited those papers?

We thank the reviewer for this important observation. We confirm that we have compared our results with relevant previous publications and have cited these articles accordingly in the Discussion section of the manuscript.

- Have you discussed the relevance and novelty of your study and what it adds to literature?

We appreciate your inquiry regarding the discussion of the relevance and novelty of our study, as well as its contributions to existing literature. We would like to clarify that these aspects are specifically addressed in the final paragraph of the Introduction section and reiterated in the Conclusion section of the manuscript and of the Abstract.

- Ensure there are the following Declarations sections at the end of your manuscript: Acknowledgements, Author contributions, Funding, Availability of data and materials statement, competing interests, Ethics approval and consent to participate.

Thank you very much for identifying the lack of information. According to your suggestion, we have included the missing Declarations sections at the end of the manuscript, prior to the References section.

# **Response to Journal Requirements**

If the reviewer comments include a recommendation to cite specific previously published works, please review and evaluate these publications to determine whether they are relevant and should be cited. There is no requirement to cite these works unless the editor has indicated otherwise.

We will comply with the reviewer's request as suggested, ensuring that the relevant publications are reviewed and evaluated for their relevance. Naturally, if it is mandatory, we will include these references.

# **Response to Reviewer #1**

Your study provides valuable evidence on the potential of the PURE-4 questionnaire to identify psoriasis patients at increased risk of developing PsA. However, several aspects require revision to strengthen the manuscript.

Thank you for your review and your comments. Our answers to your suggestions are as followed.

1. **Statistical power**: Please emphasize the small number of PsA cases (n=12 at one year) and interpret sensitivity/specificity estimates with caution, highlighting the wide confidence intervals.

Thank you for your valuable comment, which allows us to clarify our perspective. As mentioned in the fourth paragraph of the Statistical Analysis subsection of the Materials and methods section, the sample size was calculated for a precision of 5.2%, and we achieved 5.5%.

To further clarify, in the sixth paragraph of the Discussion section, we have added information about the incidence of PsA in patients with psoriasis [^[3]^]. This could help the reader to understand that the additional 5.5% reported with the PURE-4 questionnaire in our study is higher than previously reported, and our results are quite satisfactory.

Additionally, as the reviewer points out, the confidence intervals are wide and could have been corrected with a larger sample size. Although our results provide a solid starting point for future research on this topic, and considering reviewer’s comment, we have added a final limitation to the last paragraph of the Discussion section: “*Finally, given the small number of PsA cases at one year (n=12), 95% CI are wide, and results should be interpreted with caution*”.

2. **Treatment confounding**: Discuss more explicitly how systemic and biologic therapies might have influenced PsA onset and questionnaire performance.

Certainly, as the reviewer highlights, this information was not included previously in the Discussion section as this topic was not evaluated in the study. However, as additional information, we have added the following text in the fifth paragraph of the Discussion section: "*Furthermore, although not analyzed in the present study, it is worth noting that some studies show a significant decrease in the risk of developing PsA in patients with psoriasis who receive biological treatments, concluding that the use of biological drugs should be considered at an earlier stage of treatment in patients who have significant risk factors for PsA [^[4]^,^[5]^].*"

3. **Comparison with existing tools**: Include a discussion of how PURE-4 compares with other screening questionnaires (e.g., EARP), addressing advantages and limitations.

A recent systematic review of all questionnaires indicated that PURE-4 is quite a simple questionnaire with poor development [^[6]^]. Therefore, the present study attempted to address these shortcomings by adapting it to Spanish and validating it culturally and linguistically [^[7]^]. The validation methodology is explained in detail in a previously published article [2], and its performance was subsequently studied after one year in the present article.

Furthermore, another publication later recommended the use of the PURE-4 questionnaire due to its simplicity [^[8]^]. Therefore, the present study carried out the entire adaptation and validation of the questionnaire following a harmonized process [2,7].

In line with the reviewer's comments, in the fourth paragraph of the Discussion section we have incorporated an explicit comparison of the PURE-4 questionnaire with EARP (sensitivity, specificity and feasibility).

4. **Study design limitation**: Acknowledge that administering the questionnaire only once precludes assessment of reproducibility or changes in score over time.

Thank you for your insightful observation. We agree with the reviewer's comment, and for this reason, we have completed the study's limitations paragraph by adding the text "*Consequently, administering the questionnaire only once limits the ability to evaluate the reproducibility of the score over time.* *This is the first time the performance of the PURE-4 questionnaire has been tested for detecting PsA with a one-year interval between its administration and diagnosis. This fact is significant because it is recommended to screen psoriasis patients once a year (ideally, every six months), and the PURE-4 serves as a screening aid for PsA, although it does not absolve the responsibility of evaluating the patient, including their risk factors.*" to the last paragraph of the Discussion section.

Addressing these points will improve the clarity, robustness, and overall impact of your work.

Thank you very much for your thoughtful feedback. We completely agree that addressing these points significantly enhances the clarity, robustness, and overall impact of our manuscript. We appreciate your careful review and constructive suggestions, and we will ensure that each of these aspects is included in the updated version of the manuscript.

# **Response to Reviewer #2**

The study objectives are interesting and relevant for the early detection of psoriatic arthritis. The article is carefully written and methodology is robust. I have provided my comments below.

Thank you for taking the time to review our work. Please see below our answers in blue to your comments and questions.

***Abstract:***

1. Please restructure the ‘materials and methods’ part in the abstract in order to clarify to the readers that the patients with psoriasis (without previous diagnosis of PsA) from Assessment I took part in Assessment II. Please distinguish which cross-sectional studies the assessments are related to. Information on the sensitivity and specificity analysis will improve the results section further.

We appreciate your valuable observation. Following your comment, we have revised and completed the Materials and methods subsection of the Abstract to clarify this aspect and improve the reader's understanding.

***Introduction:***

2. As the validation study of the 'PURE-4 scale' was conducted in a sample from France between 2012 and 2014, the following sentence needs to be updated: “Although there are currently various tools for screening for PsA, neither a development methodology nor validation in routine clinical practice in Spain has been carried out at all times [11].” I understand that you indicated its necessity for the Spanish sample. Please make it clear for the reader. (Page 4, line 79)

Currently, several tools are available for the screening of psoriatic arthritis (PsA); however, not all of them have undergone cultural and linguistic adaptation into Spanish, nor have they been validated through a structured development methodology within routine clinical practice in Spain. Given that this is the information the authors want to reflect, and in order to make it clearer and more understandable for the reader, we have made some changes to the sentence included in the third paragraph of the Introduction section.

Furthermore, the authors aim to reinforce the work conducted and presented in the current manuscript regarding the PURE-4 tool’s ability to detect PsA, not necessarily at present, but with a projected outlook of one year.

3. In the Introduction part, in addition to the prevalence data, please add a short description on the disease and how early detection helps in reducing disease burden. I recommend expanding the literature review a little bit and include more recent studies on this topic.

We appreciate your valuable suggestions. As per your request, we have included a description of the disease and the benefits of early detection in the first two paragraphs of the Introduction section. Additionally, we have expanded the literature review to incorporate more studies on this topic.

These changes have required renumbering the references throughout the document, and this has been done without track changes.

4. I also recommend shortly reviewing the previous studies and describing a clear research gap.

Again, thank you for your valuable feedback. Please refer to our response to question 2 in this section and the changes indicated there.

***Methods:***

5. Which software was used to calculate the performance of the PURE-4 questionnaire for the early detection of potential PsA in terms of sensitivity, specificity, positive predictive value, negative predictive value? (Page 6, line 132). Discuss in brief what the term ‘area under the receiver-operating characteristic (ROC) curve’ indicates about the quality of the questionnaire.

As explained in the last sentence of the Statistical analysis subsection of the Materials and methods section: “*The statistical software Statistical Analysis System (SAS) Enterprise Guide version 7.15 was used.*”

On the other hand, we thank the reviewer for noticing the lack of information about the area under the receiver-operating characteristic (ROC) curve. Now, we have included information about its relevance in the second paragraph of the Statistical analysis subsection of the Materials and methods section, specifically: “*The area under the receiver–operating characteristic (ROC) curve with its 95% CI was calculated to assess the capacity or quality of the PURE-4 questionnaire to identify patients with PsA, considering the final diagnostic confirmation performed by the rheumatologist.*” Including this text has required restructuring the abbreviation “ROC”: previously, it was defined later in the document, but now it is defined at this point.

6. Please refer to the Youden index and describe it briefly.

Thank you again for noticing it. Considering it, we have included information about the Youden index in the third paragraph of the Statistical analysis subsection of the Materials and methods section, specifically: “*To select optimal cut-off score, the Youden index was calculated. The cut-off score associated with the highest index is considered to indicate the optimal cut-off point. Youden index is the maximum vertical distance or difference between the ROC curve and the diagonal or chance line and occurs at the cut point that optimizes the biomarker's differentiating ability when equal weight is given to sensitivity and specificity.*”

***Results:***

7. The numbers presented in the results section (Page 7, line 165) is different from what is presented in Figure 1. For example, the figure shows that Dx: no PsA (n = 223) and Dx: not evaluable (n= 11) participated in Assessment II. Shouldn’t be it 234 instead of 219? Please present identical values and check for any mistakes. In figure 3, please present the information on the participants who were not evaluable.

We thank the reviewer for appreciating it and we agree with her/him that the numbers included in the text and in the figures seem different. For this reason, we have made comprehensive modifications to Figure 1 and Figure 3 to ensure they are aligned with the text to enhance readability and comprehension. Additionally, this change has necessitated modifying the titles and captions of both figures.

8. There are differences in the values between Table 1 and S2 Table. Please check for any mistakes. If necessary, please update the title of the tables.

Thank you very much for identifying the errors.

All clinical and demographic data were collected at baseline (both tables).

In Table 1, we described baseline characteristics of evaluable patients with psoriasis not diagnosed with PsA after 1 year (included in Assessment II: n= 219). Then, in Table S2, we described baseline characteristics of all evaluable psoriasis patients who attended Assessment I and/or Assessment II (n = 253).

According to this distinction, and to facilitate the understanding of the differences between the two tables, we have modified the titles accordingly to our previous explanation.

Additionally, we have reviewed the data, corrected the detected errors, and relocated the table footnotes in both tables to improve readability

9. How Dactylitis, Enthesitis, inflammatory back pain, peripheral joint pain, DLQI, etc. were evaluated? Please describe in short in the methods section.

We would like to thank the reviewer once again for his/her valuable comment. In line with his/her observation, we have supplemented the phrase "*Descriptive analyses of all the variables were performed separately*" in the sixth paragraph of the Statistical analysis subsection of the Materials and methods section to specify which data are described in the analysis, including those mentioned by the reviewer.

***Discussion:***

10. What were the sensitivity and specificity of the original validation study for the PURE-4 scale? Please compare with your results.

Audureau E, et al. [^[9]^], showed that “*A total score (the PURE-4) was computed (0-4 points) that demonstrated excellent discriminative power (AUC = 87.6%; Sensitivity = 85.7% and Specificity = 83.6% at the threshold of ≥1/4 points), with no evidence for over-optimism in bootstrapped internal validation*” and “*At the threshold of ≥1 positive item, the resulting instrument (PURE-4) yielded both excellent sensitivity (85.7%) and speciﬁcity (83.6%)*”. However, this study does not detail the calculation of the sample size, nor did it conduct an external validation.

This information has been included in the fourth paragraph of the Discussion section to allow a comparison with the results of the present study.

11. Please discuss which relevant questions of the questionnaire were answered one year before the PsA were identified during assessment II. How does it determine the performance of PURE-4 questionnaire for early detection of PsA? If Figure 4 includes data only from assessment II (e.g., 91.7% participants with PsA diagnosis had peripheral joint pain with swelling), how can it be stated that it has assisted in early detection of PsA if it is done during the same assessment? You presented related results (both from two assessments) in S2 Fig. Please update the discussion to make it clear to the readers.

The main objective of the study was to validate the Spanish version of the Psoriatic arthritis UnclutteRed screening Evaluation (PURE-4) questionnaire in terms of sensitivity, specificity, feasibility, reliability, and construct validity, while the fourth secondary objective was to evaluate the PURE-4 questionnaire for early detection of potential PsA.

In this regard, we have created Figure 4 to describe and compare the results of the four questions of PURE-4 among patients who were ultimately diagnosed with PsA within 1 year (Assessment II):

On the other hand, in S2 Figure of the Supporting information section, we compare the results of the four questions of PURE-4 among patients who were diagnosed with PsA within 1 month (Assessment I) and within 1 year (Assessment II):

What we found in Figure S2 was that the percentage of responses to the 1^st^ and 3^rd^ questions was similar in both assessments. Question 2 was more frequently answered by patients who currently meet PsA clinical criteria, while question 4 was more frequently answered by patients who do not have PsA in assessment I but will be diagnosed within a year.

***Tables and figures:***

12. Figure 3 is difficult to understand. ‘Psoriasis + PURE 4’ and ‘Psoriasis + PsA’ seem not to be similar to one-another.

As explained in comment 7, we thank the reviewer for appreciating it and we agree with her/him that the figure seems difficult to understand. For this reason, we have made comprehensive modifications to Figure 3 to ensure its comprehension. Additionally, this change has necessitated modifying the title and captions of the figure.

13. Please check for any dissimilarities between the calculations presented in the tables and the text in the manuscript.

We thank the reviewer for pointing it out. We have thoroughly reviewed the manuscript to check for any discrepancies between the data presented in the tables and the text. After a careful examination, we did not find any inconsistencies. We hope this addresses your concern and assures you of the accuracy and consistency of the data presented.

Also, as explained in comment 7, we have made comprehensive modifications to Figure 1 and Figure 3 and their captions to ensure they are aligned with the text and to enhance readability and comprehension.

14. Please update the footnotes for better understandability and explanation.

In consideration of the reviewer's comments, we have reviewed the footnotes of all the tables and figures and have made some changes to improve clarity and comprehension in Table 1 and Table S2.

# **Response to Reviewer #3**

We appreciate your thorough review of our work. Please find our responses to your comments and questions highlighted in blue below.

1. The background introduction of PURE-4 is too brief. Could you provide a more detailed introduction?

We thank the reviewer for appreciating it and we agree with her/him that the Introduction section of the manuscript was too brief. We have expanded the information to include more details about the disease and the inconvenience of delayed diagnosis and the importance of early diagnosis.

2. Could you list the detailed items of the PURE-4 questionnaire?

In the third paragraph of the Introduction section we explained that: “*The Psoriatic arthritis UnclutteRed screening Evaluation (PURE-4) questionnaire is a tool with only four items on signs suggestive of dactylitis, enthesitis, axial involvement, and peripheral involvement*”.

While it would indeed be interesting to define the items of the PURE-4 questionnaire in greater detail, this has already been comprehensively addressed in previous publications as the original questionnaire and its Spanish adaptation and validation [2,9,7]. Therefore, the authors do not consider it necessary to elaborate further on this matter within the current document. Instead, we have provided references to other sources where this information can be found in more detail. We hope this approach is satisfactory and appreciate your understanding.

3. It is noted that 219 participants were included in the study, but the final ROC curve was plotted with n=217. Could you explain why two people were excluded?

In Assessment II, 219 patients with psoriasis and no diagnosis of PsA were included and made a second visit to the rheumatologist. However, as explained in the Sensitivity and specificity subsection of the Results section, the sensitivity and specificity of the questionnaire were evaluated considering those patients with psoriasis in whom the presence/absence of PsA was confirmed by a rheumatologist in Assessment II (n = 217).

Two people were excluded from the ROC curve because they were not evaluable: these two patients suffered from psoriasis, but rheumatologists could not make a definitive diagnosis in Assessment II.

4. Could the ROC curve be included in the manuscript?

Please, note that the ROC curve is already included in Figure 5, titled "*Area under ROC curve in Assessment II*" However, we are attaching it here for your convenience:

Additionally, we have included information in the Materials and methods section about the relevance of this ROC area for evaluating the PURE-4 questionnaire.

5. The baseline data for psoriatic arthritis are too sparse. Could some interesting indicators be added to enrich the content?

In line with the reviewer's comments, we have added more information about PsA in the initial paragraphs of the Introduction section.

6. The manuscript mainly describes the specificity and sensitivity of PURE-4 in predicting the development of psoriatic arthritis one year later. Compared with other predictive indicators, does it have an advantage?

Certainly, we appreciate the reviewer's valuable feedback. The authors understand that the PURE-4 questionnaire is a tool to support PsA screening, but that it likely does not include all the elements that could contribute to the development of PsA, for example, the presence of psoriasis in the nails, the severity of psoriasis, inverse psoriasis, the number of special locations, the patient's current treatment, etc. Although it would be ideal to be able to weight each risk factor for developing PsA, this is done piecemeal, as before.

1. [] Cuschieri S. The STROBE guidelines. Saudi J Anaesth. 2019;13(Suppl 1):S31-4. doi: 10.4103/sja.SJA_543_18
2. [] Belinchón Romero I, et al. Validation of the Spanish Version of the PURE-4 Questionnaire for the Early Detection of Psoriatic Arthritis in Psoriatic Patients. Actas Dermosifiliogr. 2024;115(7):654-62. doi: 10.1016/j.ad.2024.02.012.
3. [] Eder L, et al. The Incidence and Risk Factors for Psoriatic Arthritis in Patients With Psoriasis: A Prospective Cohort Study. Arthritis Rheumatol. 2016 Apr;68(4):915-23. doi: 10.1002/art.39494. PMID: 26555117.
4. [] Rosenthal YS, et al. Incidence of Psoriatic Arthritis Among Patients Receiving Biologic Treatments for Psoriasis: A Nested Case-Control Study. Arthritis Rheumatol. 2022;74(2):237-43. doi: 10.1002/art.41946. PMID: 34423909.
5. [] Acosta Felquer ML, et al. Treating the skin with biologics in patients with psoriasis decreases the incidence of psoriatic arthritis. Ann Rheum Dis. 2022;81(1):74-9. doi: 10.1136/annrheumdis-2021-220865. PMID: 34281904.
6. [] Landini Enríquez VV, et al. Content validity of psoriatic arthritis screening questionnaires: systematic review. Int J Dermatol. 2020;59(8):902-14. doi: 10.1111/ijd.14821.
7. [] Belinchón I, et al. Linguistic and Cultural Adaptation to Spanish of the Screening Tool Psoriatic Arthritis UnclutteRed Screening Evaluation (PURE4). Actas Dermosifiliogr (Engl Ed). 2020;111(8):655-64. doi: 10.1016/j.ad.2020.03.004.
8. [] Urruticoechea-Arana A, et al. Psoriatic arthritis screening: A systematic literature review and experts' recommendations. PLoS One. 2021 Mar 15;16(3):e0248571. doi: 10.1371/journal.pone.0248571.
9. [] Audureau E, et al. Psoriatic arthritis screening by the dermatologist: development and first validation of the ‘PURE-4 scale’. J Eur Acad Dermatol Venereol. 2018;32(11):1950-3. doi: 10.1111/jdv.14861. PMID: 29430720.
